# High-fidelity single-spin shuttling in silicon

**DOI:** 10.1038/s41565-025-01920-5

**Published:** 2025-06-09

**Authors:** Maxim De Smet, Yuta Matsumoto, Anne-Marije J. Zwerver, Larysa Tryputen, Sander L. de Snoo, Sergey V. Amitonov, Sam R. Katiraee-Far, Amir Sammak, Nodar Samkharadze, Önder Gül, Rick N. M. Wasserman, Eliška Greplová, Maximilian Rimbach-Russ, Giordano Scappucci, Lieven M. K. Vandersypen

**Affiliations:** 1 QuTech, Delft University of Technology, Delft, the Netherlands; 2 Kavli Institute of Nanoscience, Delft University of Technology, Delft, the Netherlands; 3 Netherlands Organisation for Applied Scientific Research (TNO), Delft, the Netherlands

**Keywords:** Qubits, Quantum information

## Abstract

The computational power and fault tolerance of future large-scale quantum processors derive in large part from the connectivity between the qubits. One approach to increase connectivity is to engineer qubit–qubit interactions at a distance. Alternatively, the connectivity can be increased by physically displacing the qubits. For semiconductor spin qubits, several studies have investigated spin coherent shuttling of individual electrons, but high-fidelity transport over extended distances remains to be demonstrated. Here we report shuttling of an electron inside an isotopically purified Si/SiGe heterostructure using electric gate potentials. In a first set of experiments, we form static quantum dots and study how spin coherence decays during bucket-brigade shuttling, where we repeatedly move a single electron between up to five dots. Next, for conveyor-mode shuttling, we create a travelling-wave potential, formed with either one or two sets of sine waves, to transport an electron in a moving quantum dot. This method shows a spin coherence an order of magnitude better than the bucket-brigade shuttling. It allows us to displace an electron over an effective distance of 10 μm in under 200 ns while preserving the spin state with a fidelity of 99.5% on average. These results will guide future efforts to realize large-scale semiconductor quantum processors, making use of electron shuttling both within and between qubit arrays.

## Abstract (web summary)

Conveyor-mode spin shuttling using a two-tone travelling-wave potential demonstrates an order of magnitude better spin coherence than bucket-brigade shuttling, achieving spin shuttling over 10 μm in under 200 ns with 99.5% fidelity in an isotopically purified Si/SiGe heterostructure.

## Main

To harness the full potential of quantum computation, errors will need to be corrected faster than they appear. The requirements for fault-tolerant quantum computation in terms of redundancy and error rates are generally eased when the connectivity between the qubits is stronger^1,2^. Even then, the needed redundancy will quickly bring the number of qubits into the millions. Given the challenges in realizing large-scale monolithic quantum registers, approaches based on networks of spatially separated qubit registers that are connected by quantum links^3,4^ have gained traction.

Among the numerous quantum computing platforms, gate-defined semiconductor spin qubits^5^ have garnered considerable attention. Recent advances in this field showcase extended spin coherence^6^, high-fidelity single-^7–9^ and two-qubit^10–13^ gates, high-temperature operation^14,15^ and universal control over up to six qubits^16^. Moreover, spin qubits are an attractive choice for densely packed quantum processors because of their compatibility with existing semiconductor fabrication techniques^17,18^ and pitch of about 100 nm.

Whereas the conventional two-qubit gate relies on the exchange interaction between spins in adjacent quantum dots, several avenues for increasing the connectivity between distant spin qubits on the same chip have been explored. Much effort has gone into engineering hybrid devices where superconducting resonators are used to couple electron or hole spins in quantum dots. This culminated in the recent observation of iSWAP oscillations between two spins separated by a few hundred micrometres^4^, swapping their states and adding a phase of i to the ∣01〉 and ∣10〉 components. A promising alternative for distances of up to about 10 μm consists in transporting (shuttling) spins across the chip, which can increase the connectivity within a qubit register and form a coherent link between qubit registers^19–24^. Experiments performed with trapped ions^25,26^ and neutral atoms^27,28^ have similarly demonstrated physical transport of qubits.

Two distinct procedures for spin shuttling exist, referred to as bucket brigade and conveyor mode. Bucket-brigade shuttling involves transporting a spin through an array of quantum dots by successively adjusting their electrochemical potentials. Successful charge transfer was realized across nine dots^29^ and spin-flip probabilities per hop below 0.01% were observed in both GaAs^30^ and Si/SiGe quantum dot arrays^31,32^. Preservation of the spin phase was probed qualitatively in GaAs quantum dots^33,34^ and quantitatively in a silicon double quantum dot, with phase-flip probabilities of 0.7% (ref. ^35^) and 0.1% (ref. ^32^) per hop during a Hahn-echo sequence. Near zero magnetic field, even lower phase-flip probabilities have been reported^36^. In addition, it was shown in Ge quantum dots that diabatic bucket-brigade shuttling in the presence of spin–orbit interaction^37^ can be used to generate single-qubit gates with fidelities of 99.97% (ref. ^38^). Conveyor-mode shuttling is a technique in which a travelling-wave potential transports the spin inside a moving quantum dot. The travelling-wave potential can be generated by a surface acoustic wave or by phase-shifted sinusoidal signals applied to successive gate electrodes. Also with conveyor-mode shuttling, charge transfer was demonstrated along channels of on the order of 10 μm long^39–41^. Coherent spin transfer was studied by moving one of two electrons prepared in a spin singlet state^42,43^. However, the relative performance of the two methods has not been compared directly so far. More importantly, coherent spin transfer over extended distances that is both fast and of high fidelity remains to be demonstrated.

In this work, we quantitatively investigate the phase-flip probability when repeatedly moving an electron back and forth through a linear device in isotopically purified Si/SiGe, using Ramsey-style and Hahn-echo-style measurements. We compare the performance of bucket-brigade and conveyor-mode shuttling, including a conveyor-mode implementation that introduces sine waves with two frequencies instead of one, and study the performance as a function of the driving amplitude and shuttling speed. The conventional single-tone conveyor reaches a shuttle speed of 36 m s^−1^, while the two-tone conveyor is still successfully operated at 64 m s^−1^. Finally, we execute interleaved randomized benchmarking (RB) to quantify the shuttling fidelity for a cumulative displacement of 10 μm through the device, yielding a fidelity of 99.54 ± 0.05%.

## Device design and characterization

**Fig. 1:**
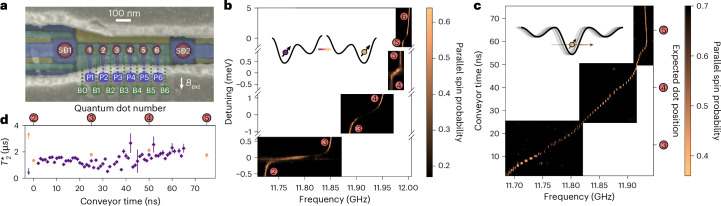
Device and characterization. **a**, A false-coloured scanning electron microscopy image of a nominally identical device to the one used in this work. The colours indicate different metallization layers. Six plunger (P_*i*_ in blue), seven barrier (B_*i*_ in green) and two screening (yellow) gates form a linear array of six quantum dots (indicated by numbered circles). Two sensing dots (SD_*i*_) are placed at both ends of the array. A cobalt micromagnet, shown in sage grey, is placed on top of the active area, and an in-plane external magnetic field (*B*_ext_) is applied. Details about the device fabrication and measurement set-up can be found in Supplementary Notes I and XV, respectively. **b**, EDSR spectroscopy of the spin resonance frequency as a function of interdot detuning for each pair of neighbouring dots. The spins in quantum dots 1 and 2 are initialized in an anti-parallel state, after which the single spin from dot 2 is shuttled in bucket-brigade mode to the respective dot pair, a microwave burst is applied at a given interdot detuning and the spin is read out after shuttling back to dot 2. The colour scale shows the parallel spin probability, which here is a measure of the spin-flip probability, as a function of the applied frequency and interdot detuning. The inset depicts bucket-brigade mode tunnelling, transferring a single electron between dots. **c**, EDSR spectroscopy of the spin resonance frequency when shuttling a single spin in (two-tone, see below) conveyor mode. The spins in quantum dots 1 and 2 are initialized in a parallel state. The single spin from dot 2 is displaced by the conveyor potential by a distance controlled by the conveyor time, subjected to a microwave burst, displaced back to dot 2 and read out. The colour scale shows the parallel spin probability as a function of the applied frequency and conveyor time (the expected conveyor displacement is shown on the right axis relative to the plunger gate positions). The inset depicts the travelling-wave potential that smoothly transfers the electron. **d**, The spin dephasing time in the gate-defined quantum dots (orange) and in a static two-tone conveyor at different conveyor times, corresponding to different locations along the channel (purple). In both cases, fits were performed with Gaussian decay and the error bars correspond to one standard deviation to the fit.

The device is fabricated on a ^28^Si/SiGe heterostructure, hosting a linear array of six quantum dots (Fig. 1a). A cobalt micromagnet is deposited on top of the gate electrodes. Its stray field enables electric-dipole spin resonance (EDSR) for single-qubit rotations^44^ and separates the spin resonance frequencies for electrons in different dots.

We place a reference electron in dot 1 and initialize an electron spin for shuttling in dot 2. All other quantum dots are emptied in order to investigate coherent spin shuttling through the array. For bucket brigade, we not only pulse the electrochemical potentials but also raise the interdot barrier gate voltages one after the other to temporarily establish a large interdot tunnel coupling *t*_c_ (between 17 GHz and 55 GHz; Supplementary Note II). A large tunnel coupling is required for a rapid transfer between dots while maintaining adiabaticity^30,33,34^. Figure 1b shows how the qubit resonance frequency abruptly shifts as an electron is displaced between neighbouring dots. For conveyor-mode shuttling we use a number of phase-shifted sine signals applied to the plunger and barrier gates. The resulting travelling-wave potential transports the electron within a single potential minimum. This technique allows continuous control of the electron position along the array. Figure 1c shows a continuous trend of the resonance frequency (albeit different than expected; Supplementary Note XIV).

The local spin dephasing time ${T}_{2}^{\;* }$ can be probed for an electron in a static conveyor potential minimum as well as in any of the predefined dots (Fig. 1d). In both cases, microwave driving is applied when the spin is in dot 2, while the idling time is spent in either a predefined dot or in a static conveyor potential minimum. ${T}_{2}^{\;* }$ shows an increasing trend towards dot 5, where the local magnetic field gradient from the micromagnet is weaker. Presumably, charge noise leads to dephasing as it modulates the electron position in the gradient magnetic field, with additional dephasing contributions from residual hyperfine noise. We note that the dephasing times in the predefined dots are in general slightly longer than those in the conveyor minimum, indicating a tighter confinement and, hence, reduced electrical susceptibility in the predefined dots.

## Bucket-brigade operation

We study the bucket-brigade shuttling performance via Ramsey- and Hahn-echo-style measurements. We first investigate shuttling between adjacent dots (similar to refs. ^32,35^) and next perform shuttling across multiple quantum dots.

**Fig. 2:**
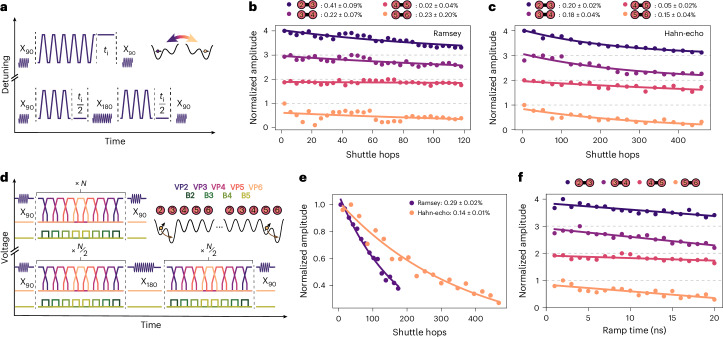
Bucket-brigade shuttling. **a**, Ramsey (top) and Hahn-echo (bottom) pulse sequences when shuttling repeatedly between sites of a double quantum dot. The detuning is pulsed in 2 ns roughly between the charge symmetric points of the (1, 0) and (0, 1) states of the double dot, where there is a wait time of 1 ns. X_90_ and X_180_ gates are applied in dot 2 with microwave pulses. **b**,**c**, Decay of the Ramsey (**b**) and Hahn-echo (**c**) fringe amplitude for each double dot with increasing number of shuttle hops, following the pulse scheme of **a**. Plots are normalized by the amplitude of the highest data point and are offset by 1 for clarity. A fitted exponential decay yields the indicated phase-flip probabilities. Performing the idling operation in different dots for the Ramsey and Hahn-echo sequences has no significant impact on the extracted phase-flip probability compared with the one-standard-deviation error bar (Supplementary Note VII). **d**, Ramsey (top) and Hahn-echo (bottom) pulse sequences when shuttling repeatedly through the array from quantum dot 2 to 6. The detunings are pulsed in 2 ns when the respective tunnel couplings are temporarily increased. The wait time per dot is 4 ns (Supplementary Note III). **e**, Decay of the Ramsey and Hahn-echo fringe amplitude with increasing number of shuttle hops, following the pulse scheme of **d** between dots 2 and 5. **f**, Normalized Hahn-echo fringe amplitude after shuttling forth and back twice through a double dot as a function of the ramp time. The uncertainties correspond to one standard deviation extracted from the fitting procedure.

After initializing the reference spin in dot 1 and the spin in dot 2 in a parallel state, an X_π/2_ gate is applied to the spin in dot 2. Next, this spin is transported to the target double dot after which it is repeatedly transferred back and forth between the two sites (Fig. 2a). The number of shuttle hops *N* is varied while the idle time *t*_i_ at the end of the shuttle sequence is adjusted, keeping the total sequence time constant. This allows one to isolate the impact of hopping on the spin coherence. In the Hahn-echo measurements, a refocusing X_π_ gate is applied in dot 2 after half the number of hops. After shuttling, a second X_π/2_ rotation is applied in dot 2 before the spin state is measured using parity readout.

Figure 2b and Fig. 2c show the decaying normalized amplitude of Ramsey and Hahn-echo fringes, respectively, with increasing number of shuttle hops. The fringes are measured in a rotating frame that is detuned from the qubit frequency by about 30 MHz. For all four double dot pairs, the decay is fitted to an exponential function *a* (1 − *ϵ*)^*n*^, where *n* is the number of shuttle hops and *ϵ* is the coherence loss per hop. We here define phase-flip probability $\frac{\epsilon }{2}$ as the probability for the spin to undergo a phase-flip error during shuttling, excluding initialization and measurement errors. This yields phase-flip probabilities below 0.5% per hop (dividing the total error by the number of hops) and in some cases as low as 0.02%. The phase-flip probability is lower in double dots with a smaller Zeeman splitting difference (Fig. 1b), which agrees with numerical simulations (Supplementary Note V). Double dot 5–6 does not follow this trend, which is possibly related to the comparatively large voltage needed on gate B5.

Next, we evaluate spin coherence during sequential shuttling through the entire array. We pulse both the interdot detuning and the tunnel coupling between the successive double dots in turn. Pulsing the tunnel couplings enables us to reach a high tunnel coupling between the target dots to ensure adiabatic transport, while suppressing charge leakage to adjacent dots by reducing the corresponding tunnel couplings (Fig. 2d). Figure 2e shows the decaying amplitude of Ramsey and Hahn-echo fringes, respectively, with increasing number of shuttle hops between dot 2 and dot 5 (bucket-brigade shuttling works best between these dots; Supplementary Note VI). In contrast to the shuttling benchmark in double dots (Fig. 2b,c), we vary the number of hops without additional idle times at the end of the shuttling sequence. This means that spin coherence lost during the shuttling process or while idling in individual dots cannot be distinguished. However, this approach does reveal how spin coherence is affected overall when shuttling under realistic conditions across many quantum dots. We obtain an average phase-flip probability per hop of 0.29 ± 0.01% with the Ramsey protocol and of 0.14 ± 0.01% with the Hahn-echo protocol.

The spin dephasing time ${T}_{2}^{\;* }$ extracted from the bucket-brigade shuttling measurements in Fig. 2e is 1.04 μs, compared with an average ${T}_{2}^{\;* }$ of 1.75 μs in the static dots. This indicates that the act of hopping between dots increases the phase-flip probability. As stated in other works^35,37,45,46^, charge noise couples more strongly to the qubit in the low detuning regime of each double quantum dot, where the resonance frequency is highly sensitive to detuning fluctuations (Fig. 1b). Phase flips from hopping can also occur if the charge transitions are not perfectly adiabatic with respect to the tunnel couplings (Supplementary Note IV). Small uncertainties in the timing of charge transfer then lead to dephasing due to the Larmor frequency difference between the quantum dots. Such diabatic transitions can in principle result either from the high-frequency components of the voltage ramp or from rapid electric field fluctuations arising from charge noise. Figure 2f shows that the phase-flip errors monotonously decrease with decreasing ramp time down to 2 ns. Furthermore, the loss of phase coherence increases with the Zeeman splitting difference. These observations are consistent both with enhanced dephasing halfway the interdot transition (see the simulations in Supplementary Note V) and with diabatic transitions from rapid electric field fluctuations as the phase-flip mechanism.

## Conveyor-mode operation

**Fig. 3:**
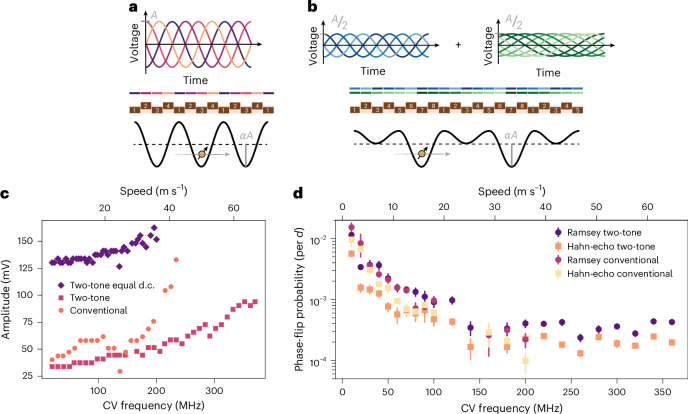
Conveyor-mode shuttling. **a**, Oscillating gate voltages (top) and the associated travelling-wave potential in the quantum well (bottom) for the conventional four-phase conveyor. Gates can be divided into four sets (numbered 1–4), and the colour above each gate corresponds to the phase of the applied sine signal. *A* indicates the amplitude of the voltage signal, and the lever arm *α* expresses the conversion of gate voltage to dot potential. In the experiment, two different amplitudes are applied to gates in separate fabrication layers to compensate for the difference in lever arm (not shown in the figure for simplicity). **b**, Gate voltages as a sum of two oscillating signals with frequencies *f* and *f*/2 (top) and the associated travelling-wave potential in the quantum well (bottom) for the two-tone conveyor. Gates can be divided into eight sets (numbered 1–8), and the colours above each gate correspond to the phases of the applied sine signals. Each sine wave uses half the amplitude of the four-phase conveyor. **c**, The minimal conveyor (CV) amplitude required for successful charge transfer by shuttling, accounting for the known attenuation in the transmission lines (see Supplementary Note XIII for a figure accounting for the filter function built into the arbitrary waveform generator as well). The conveyor potential minimum is displaced from below P2 to below P5, a resonant microwave burst is applied to flip the spin using EDSR (if the electron was successfully transferred), the potential minimum is moved back to below P2, and the electron spin is read out. Individually adjusted d.c. voltages are used in the orange and pink cases (Supplementary Note III). For the purple data points, a common plunger and a common barrier d.c. voltage is applied. The shuttling speed is calculated using the applied conveyor frequency and the 180-nm wavelength set by the gate pitch. **d**, Phase-flip probability per nominal *d* = 90 nm plunger-to-plunger distance for the conventional conveyor and the two-tone conveyor as a function of the applied conveyor frequency. All conveyor amplitudes are set to *A* = 100 mV, except for the two-tone conveyor amplitudes above 200 MHz, which are set to 120 mV. The need to increase the amplitude can be understood from **c**. For conveyor frequencies between 100 MHz and 160 MHz, spin coherence in the conventional conveyor is completely lost (see the missing data points). While the underlying reasons for this phenomenon remain unclear, we provide additional comments in Supplementary Note X. The phase-flip probabilities are extracted from the best fit of the exponential decay of Ramsey or Hahn-echo fringes with increasing shuttling distance. A comment on the data analysis can be found in Supplementary Note XVI. The error bars correspond to one standard deviation extracted from the fit.

Turning to conveyor-mode shuttling^43,47,48^, the traditional approach makes use of four phase-shifted voltage signals applied to a set of gate electrodes, with the voltage

1 $${V}_{n}(t)={V}_{n}^{\;{\text{d.c.}}}-A\sin \left(2\uppi ft-{\phi }_{n}\right)$$

applied to gate *n*, where ${\phi }_{n}={\phi }^{{\prime} }+(n\,\mathrm{mod}\,\,4)\,\uppi /2$ and ${\phi }^{{\prime} }$ is a phase offset that determines the initial position of the potential minima. Here, ${V}_{n}^{\;{\text{d.c.}}}$ represents a d.c. voltage offset and *f* is the conveyor frequency. In most measurements shown below, we individually adjust the d.c. voltage applied to each gate. The voltage signals and resulting travelling potential wave are illustrated in Fig. 3a.

We additionally propose and assess a novel two-tone conveyor approach with voltage signals given by

2 $${V}_{n}(t)={V}_{n}^{\;{\text{d.c.}}}-\frac{A}{2}\left[\sin \left(2\uppi ft-{\phi }_{n}\right)+\sin \left(\uppi ft-{\theta }_{n}\right)\right].$$

This conveyor incorporates a second sine wave with exactly half the original conveyor frequency and ${\theta }_{n}={\phi }^{{\prime} }/2+(n+1\,\mathrm{mod}\,\,8)\,\uppi /4$. While requiring twice the number of distinct control signals compared with the conventional conveyor, a notable advantage in the shape of the travelling potential wave (Fig. 3b) is achieved. Destructive interference at every second potential minimum strongly suppresses charge leakage to neighbouring moving dots during shuttling. This is especially relevant in the presence of disorder in the background potential landscape. In equations (1) and (2), we have assumed that equal amplitude sine signals are applied to all gates. Given that the barrier gates are less well coupled to the channel than the plunger gates, the amplitude applied to the barrier gates was 1.4 times that applied to the plunger gates, a ratio that we found works well (all values below refer to the plunger gate amplitudes). We note that also the two tones could be applied with different amplitudes.

To determine the amplitude required for successful charge transfer by the conveyor, we shuttle a single spin from below gate P2 to close to below gate P5, apply an X_π_ gate and shuttle back. We then identify the minimal conveyor amplitude for which the spin flip from the X_π_ gate is detected at readout, indicating successful charge transfer (the transition typically occurs within a few millivolts). Figure 3c shows the minimal amplitude as a function of the main conveyor frequency, where we observe that the conventional conveyor necessitates a higher amplitude than the two-tone conveyor. The data suggest that even faster transport would be feasible, especially for the two-tone conveyor (the shuttling speed was limited by the output filter of the control hardware). For comparison, we also show the minimum amplitude for the two-tone conveyor when the same d.c. voltage is applied to all plunger gates, and another fixed d.c. voltage is applied to all barrier gates^41,43,48^. Operating a conveyor with equal d.c. voltages applied to all gates would reduce the overhead involved in operating long-distance shuttling channels. We see that, when the disorder in the background potential landscape is not compensated for by the local d.c. voltages, we can still successfully displace charges using the conveyor, albeit requiring a higher conveyor amplitude.

We characterize coherent spin transport in conveyor mode by recording the phase-flip probability in Ramsey- and Hahn-echo-style sequences, using similar methods as used for bucket-brigade shuttling. Figure 3d shows the phase-flip probability, defined per plunger-to-plunger distance *d* to allow comparison with bucket brigade, for both conveyor types. Initially the probability decreases with increasing speed. In general, faster transfer means the spin has less time to dephase while it is transported in the moving dot. This behaviour follows the prediction by ref. ^47^. Above 150 MHz, the measured phase-flip probability does not keep decreasing but saturates, for reasons that are not fully understood. The Ramsey phase-flip probabilities are similar between the conventional and two-tone conveyor, whereas the echo sequences show better results for the two-tone version. Importantly, the two-tone case allows considerably higher speeds with modest conveyor amplitudes (Fig. 3c). Even though the observed phase-flip probabilities level off above 150 MHz, faster shuttling is still advantageous in future scenarios where a subset of qubits is subject to decoherence while others are shuttled.

The data pertaining to dephasing in a moving conveyor are well fitted to a single exponential, indicating high-frequency components in the noise experienced by the spin. For the static (two-tone) conveyor, the decay is close to Gaussian, pointing at low-frequency noise dominating the decay. This suggests that the exponential decay in a moving conveyor results primarily from moving through a spatially varying but quasi-static noise environment. Moreover, the extracted (exponential) dephasing time for both the conventional and two-tone conveyor during shuttling is longer than the (Gaussian) decay time in the static two-tone conveyor case in Fig. 1d (Supplementary Note IX). This indicates that shuttling in conveyor mode does not suffer from important additional dephasing channels and in fact may benefit from motional narrowing effects^47,49^. We note there is no clear dependence of ${T}_{2}^{\;* }$ on conveyor speed, which would suggest that a motional narrowing effect in this finite-size device is already fully present at the slowest shuttling speeds, giving rise to an increase in dephasing times.

## Shuttling fidelity

**Fig. 4:**
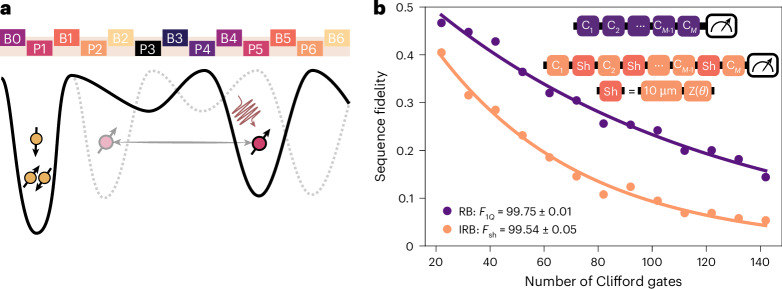
Shuttling fidelity. **a**, A schematic of the potential landscape when performing interleaved RB for repeated shuttling of a single spin using a two-tone conveyor. Quantum dot 1 hosts three electron spins serving as an ancilla for Pauli-spin blockade readout. Specifically, we use a 300 MHz conveyor frequency, displace the electron for 4 ns and move it back in 4 ns, and repeat this over 24 rounds, corresponding to a cumulative distance of 10 μm, which takes 184 ns. Single-qubit control with microwave pulses is always performed with the electron roughly under gate P5. **b**, Single-qubit RB (purple) as a reference for the shuttling gate interleaved RB (IRB) (orange). The solid lines show the best-fit parameter values for an exponential fit. Here, the uncertainties in the gate fidelities represent one standard deviation and are obtained by bootstrap resampling^53^. The inset shows the circuit for the single-qubit reference (purple) and 10 μm shuttling interleaved RB (orange), where *C*_*i*_ are single-qubit Clifford gates. The shuttling operation consists of moving the electron back and forth over an accumulated 10 μm distance, and a virtual Z phase compensation. These RB data were acquired with the superconducting magnet in persistent mode, contrary to the measurements presented in all previous figures. Shuttling interleaved RB data with the magnet in driven mode, along with Ramsey measurements, can be found in Supplementary Note XI. Interleaved RB data for shuttling with equal d.c. voltages can be found in Supplementary Note XII.

Finally, we characterize the shuttling fidelity of the high-speed two-tone conveyor by using RB. Figure 4a shows a schematic of the experiment. The spin is initialized in dot 2; then the conveyor potential is switched on and the electron is shuttled to below gate P5, where we execute Clifford operations using EDSR with the electron in the static conveyor minimum (in this way, we avoid potential errors in transferring the spin from a fixed dot potential to a conveyor minimum). To benchmark the shuttling fidelity, we compared the decay of a standard RB sequence with that of an interleaved RB sequence. We interleave repeated conveyor shuttling operations, roughly between gates P2 and P5, followed by a virtual Z operation that ensures that the total operation (ideally) corresponds to the identity gate. For this part of the experiment, we further optimize the pulsed offset voltages applied to the plunger and barrier gates during shuttling using a global optimizer^50^. In addition, we operate the magnet in persistent mode instead of in driven mode, which results in twice longer ${T}_{2}^{\;* }$ values. Figure 4b shows the results for both the reference and interleaved RB sequence in this regime. We obtain a single-qubit gate fidelity *F*_1*Q*_ of 99.75 ± 0.01% and a shuttling fidelity *F*_sh_ of 99.54 ± 0.03% for shuttling over a distance of 10 μm.

Our analysis indicates that the conveyor shuttling fidelity is limited mainly by the ratio between the shuttling time and the static ${T}_{2}^{\;* }$. Additional improvements in the coherence time can be achieved by reducing charge noise and minimizing magnetic field gradients. Remarkably, the obtained *F*_sh_ already corresponds to a shuttle fidelity of 99.996% over a plunger-to-plunger distance, and the fidelity decays by 1/e (with e the base of the natural logarithm) after about 2.25 mm, if we simply extrapolate exponentially.

## Conclusions

This work demonstrates coherent bucket-brigade shuttling in silicon across multiple dots. We obtain phase-flip probabilities below 0.2% per hop when shuttling back and forth through the array. Conveyor-mode shuttling, using both conventional four-phase and novel two-tone conveyors, exhibits stable and highly coherent spin transport, with phase-flip probabilities about one order of magnitude lower than our best bucket-brigade results for a comparable distance. Using a two-tone conveyor, we are able to shuttle an electron back and forth over a cumulative distance of 10 μm in less than 200 ns and with a fidelity of 99.5%.

In this comparative investigation of shuttling methods, we find that conveyor-mode shuttling allows faster spin transfer with a higher fidelity, limited by the inherent dephasing in the device. It avoids sequential adiabatic interdot crossings and repeated charge delocalization intrinsic to bucket-brigade shuttling. For successful conveyor-mode operation, we must ensure the electron does not escape from the travelling potential minimum, which can be especially challenging in the presence of disorder. With only a limited increase in control parameters, the two-tone conveyor reduces this escape probability and we find it allows higher shuttling speeds with lower drive amplitudes.

We note that future devices would probably not utilize a micromagnet on top of the shuttling channel, which could lead to even higher shuttling fidelities as the Zeeman splitting gradients will be orders of magnitude smaller. Moreover, one can encode the qubit in the *S*–*T*^0^ subspace and shuttle both electrons sequentially to further protect the shuttled information against quasi-static noise^19^. However, whereas the valley splitting in this device is estimated to be above 170 μeV (Supplementary Note VIII), the possibility of encountering local regions with low valley splitting increases for longer quantum links^51^. This would require one to (locally) slow down the conveyor^47^ in order to avoid detrimental valley excitations, which would degrade the shuttling fidelity. Furthermore, we note that the best results are obtained when tweaking the individual d.c. gate voltages. Future devices with reduced control complexity should aim at high-fidelity spin shuttling along a 1–10-μm-long channel using a small number of interleaved comb-shaped gates to propagate electrons. Broadly speaking, it is desirable for the shuttling error rates to be well below the two-qubit gate error rates, which will necessitate a low level of background potential disorder^52^.

## Methods

### Device, initialization and readout

The device is fabricated on an isotopically purified ^28^Si/SiGe heterostructure featuring a 7-nm quantum well. The confinement potentials are defined by three layers of Ti:Pd gates separated by Al_2_O_3_, serving as screening, plunger (P) and barrier (B) gates, respectively. Sensing dots are placed at the ends of the linear array to facilitate charge sensing and to act as electron reservoirs. An external magnetic field of 260 mT is applied in the plane of the quantum well, and all experiments are performed in a dilution refrigerator set to a temperature of 200 mK (ref. ^14^).

We operate at the (3, 1)–(4, 0) charge transition of dots 1 and 2, creating a sizable readout window for parity Pauli-spin blockade^16,54^. Initialization of two electron spins is done by ramping from the (4, 0) to the (3, 1) charge state, subsequently performing parity readout of the spin states and post-selecting the single-shot runs with either the parallel or anti-parallel measurement outcomes.

### Shuttling interleaved RB

We analyse the shuttling operation fidelity using interleaved RB. For the reference sequence, we perform conventional RB based on randomly selected sequences of Clifford operations. We measure the probability for the final spin state to correspond to the initial spin-up or spin-down state as a function of the number of Clifford operations. The initial state is selected by an optional microwave burst before the RB sequence. The final step involves subtracting the measured probabilities for the two initial states from each other to minimize the uncertainty associated with the exponential fitting of the data. As the number of applied Clifford gates *N* increases, the return probability decreases. We fitted this decay to ${F}_{{\rm{seq}}}=A{p}_{{\rm{c}}}^{N}$, where *p*_c_ is the depolarizing parameter and the amplitude *A* depends on state preparation and measurement errors. The average fidelity per Clifford operation *F*_c_ and per single primitive gate ${F}_{{\rm{c}}}^{{\rm{single}}}$ is then calculated using

3 $${F}_{{\rm{c}}}=(1+{p}_{{\rm{c}}})/2,$$

4 $${F}_{{\rm{c}}}^{\;{\rm{single}}}=\frac{1+{p}_{{\rm{c}}}^{{\rm{single}}}}{2} \sim 1-\frac{1-{F}_{{\rm{c}}}}{1.875}.$$

We estimate the fidelity for coherently shuttling of a spin over about 10 μm by interleaved RB. In between successive Clifford operations, the electron is shuttled back and forth repeatedly over a distance of 10 μm. We call the depolarizing parameter for the interleaved sequence *p*_sh_. The fidelity for shuttling over 10 μm *F*_sh_ is then estimated as

$${F}_{{\rm{sh}}}=\frac{1+\left(\;{p}_{{\rm{sh}}}/{p}_{{\rm{c}}}\right)}{2}\ .$$

The uncertainties in the gate and shuttling fidelities are estimated using a bootstrap resampling^53^. By repeating the resampling process 10^4^ times, we obtain distributions of the single-qubit gate fidelity ${F}_{{\mathrm{c}}}^{{\rm{single}}}$ and the shuttling fidelity *F*_sh_. The final uncertainties are calculated as the standard deviations of these distributions.

The total effective shuttling distance *D*_sh_ in Fig. 4 is calculated as

5 $${D}_{{\mathrm{sh}}}=2N\,{f}_{{\mathrm{CV}}}\,{t}_{{\mathrm{sh}}}\,2d.$$

Here, *N* is the number of shuttle rounds, *f*_CV_ is the conveyor frequency and *t*_sh_ is the one-way shuttle time. Note that 2*d* is the shuttled distance per conveyor period, which is double the plunger-to-plunger distance *d*. Given 24 shuttle rounds at a 300 MHz conveyor frequency, with *t*_sh_ = 4 ns and 2*d* = 180 nm, *D*_sh_ yields 10.368 μm. We note that local disorder in the potential landscape at the boundaries of the shuttle trajectory could be a limiting factor for the accuracy of this shuttle distance estimate.

## Online content

Any methods, additional references, Nature Portfolio reporting summaries, source data, extended data, supplementary information, acknowledgements, peer review information; details of author contributions and competing interests; and statements of data and code availability are available at 10.1038/s41565-025-01920-5.

## Supplementary information

Supplementary Information Supplementary Notes I–XVI, Figs. 1–12 and Tables I and II.

## Data Availability

The data supporting this work are available via Zenodo at 10.5281/zenodo.10834810 (ref. ^55^). Other raw and relevant data from the study are available from the corresponding authors upon reasonable request.

## Code availability

The codes used for data processing and numerical simulation are available via Zenodo at 10.5281/zenodo.10834810 (ref. ^55^).
