# Supplementary material for: High-fidelity single-spin shuttling in silicon
Source: Nat Nanotechnol. 2025 Jun 9;20(7):866–72. doi: 10.1038/s41565-025-01920-5 (PMC12267049; doi:10.1038/s41565-025-01920-5)
Supplement: Supplementary file 1 — Supplementary Notes I–XVI, Figs. 1–12 and Tables I and II. [file 41565_2025_1920_MOESM1_ESM.pdf]

nature nanotechnology

Supplementary information

https://doi.org/10.1038/s41565-025-01920-5

High-fidelity single-spin shuttling in silicon

In the format provided by the authors and unedited

This supplementary information includes:

- Supplementary Note I Device fabrication
- Supplementary Note II Lever arms and tunnel couplings for bucket-brigade shuttling
- Supplementary Note III Gate voltage conditions during shuttling
- Supplementary Note IV Considerations on noise sources
- Supplementary Note V Simulation of dephasing during bucket brigade shuttling
- Supplementary Note VI Bucket-brigade shuttling from quantum dot 2 to 6
- Supplementary Note VII Idling site for double dot bucket-brigade shuttling
- Supplementary Note VIII Valley splitting
- Supplementary Note IX Dephasing time during conveyor shuttling
- Supplementary Note X Data gap for the 100-160 MHz conveyor frequency band
- Supplementary Note XI Two-tone conveyor shuttling fidelity with driven external electromagnet
- Supplementary Note XII Two-tone conveyor shuttling fidelity with equal DC voltages
- Supplementary Note XIII Required conveyor amplitude corrected for AWG filter
- Supplementary Note XIV Micromagnet simulation
- Supplementary Note XV Measurement set-up
- Supplementary Note XVI Data analysis of Ramsey- and Hahn-echo-like shuttling measurements

I. DEVICE FABRICATION

The device used in this work is fabricated on a $^{28}Si/SiGe$ heterostructure [1]. Supplementary Figure 1 depicts a cross-section of the active area of the device. First, a 1.5 µm linearly graded $Si_{1-x}Ge_x$ buffer is grown on a Si wafer. On top of that, a relaxed 300 nm thick $Si_{0.7}Ge_{0.3}$ spacer is grown, following by a 7 nm isotopically purified (800 ppm) tensile-strained ^{28}Si quantum well [2]. Another 30 nm $Si_{0.7}Ge_{0.3}$ spacer passivated with dichlorosilane at 500 °C [3] separates the quantum well from the gate stack. Ohmic contacts to the two dimensional electron gas in the QW are made using phosphorus-ion implantation. A 10 nm Al_2O_3 layer precedes three layers of Ti:Pd deposited using electron beam evaporation. The Ti:Pd gate layers have a thickness of 3:17, 3:27, 3:37 nm, respectively, and are separated by 5 nm thick Al_2O_3 layers deposited by atomic layer deposition. Finally, another 5 nm thick Al_2O_3 layer is deposited on top of the gate stack, followed by a 5:200 nm thick Ti:Co micromagnet, used for addressing and driving of the qubits.

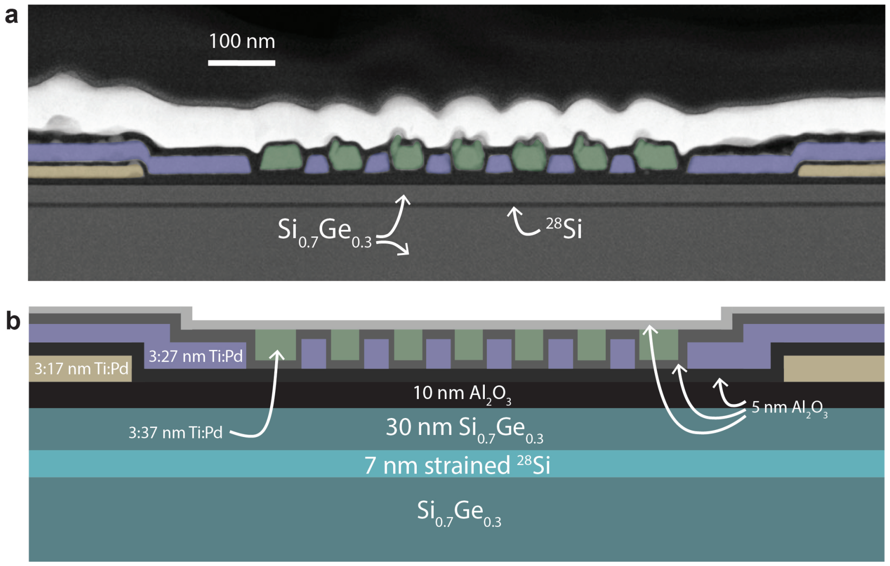

Supplementary Figure 1. Cross-section of a nominally identical device before deposition of the cobalt micromagnet. a) False-colored transmission electron microscope (TEM) cross-section image of the active area of the device. Eight (sensing) dot plunger (blue), seven barrier (green) and two screening (yellow) gates are visible. The white material on top of the device is a Pt cap added for improved imaging. Light grey areas in the Al_2O_3 do not correspond to electrical shorts between metallization layers, but are caused by local topography that is averaged across the thickness of the TEM lamella. b) Schematic representation of the cross-section, also indicating the different oxide layers.

II. LEVER ARMS AND TUNNEL COUPLINGS FOR BUCKET-BRIGADE SHUTTLING

For bucket-brigade shuttling, the tunnel couplings between the dots are crucial for the shuttling performance. Here we estimate the lever arms of the virtual plunger gates and use them to extract the tunnel couplings between the quantum dots. Supplementary Figure 2a shows the polarization line of the first electron in dot 6 at a mixing chamber temperature of 500 mK. The barrier to the reservoir is sufficiently closed such that the transition is mostly temperature broadened. This allows us to extract the lever arm α_{VP6} of virtual gate VP6 by fitting the transition line to $a\,VP6 + b + c\left(1 + e^{\alpha_{VP6}\,(VP6-d)\,\beta_e}\right)^{-1}$. Here, a, b, c and d are fitting parameters and $\beta_e = (k_B\,T_e)^{-1}$ with k_B the Boltzmann constant and T_e the electron temperature, set to 500 mK. From the slope of the respective (1,0)-(0,1) anticrossings, we extract the relative lever arms of the virtual plunger gates in each double quantum dot. This yields a lever arm value for each virtual plunger gate.

In Supplementary Figure 3, the tunnel coupling t_c between each quantum dot is obtained by fitting the spin resonance frequency at the interdot transition to $a\,(\epsilon - \epsilon_0) + b + c\,\frac{\epsilon-\epsilon_0}{\sqrt{(\epsilon-\epsilon_0)^2+4\,t_c^2}}$, where ϵ is the double dot detuning and a, b, c, ϵ_0 and t_c are fitting parameters. This corresponds to a simplified picture [4], where we assumed that the tunnel coupling is large compared to the Zeeman splitting, and that spin-dependent tunneling and the Stark shift difference between the dots are negligible.

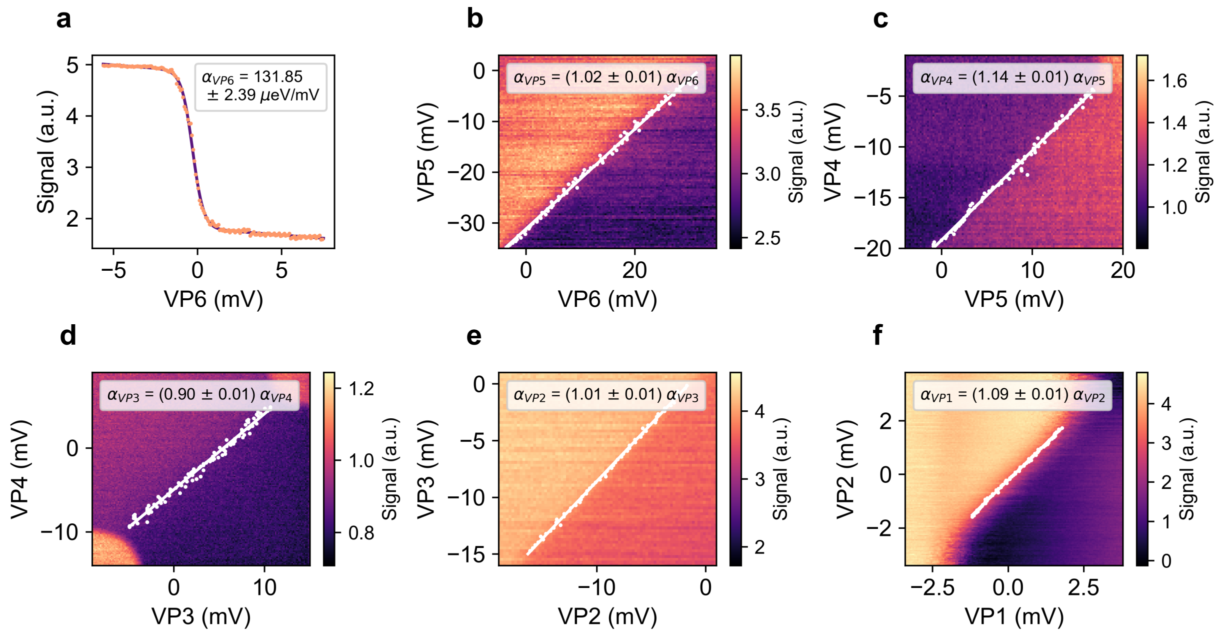

Supplementary Figure 2. **Lever arm analysis.** a) Charge transition for dot 6. b)-f) Charge stability diagram near the interdot transition, showing a charge detector signal as a function of two virtual gate voltages. The transition is extracted for each horizontal scan (white points) and the ratio of the lever arms of the two virtual gates is estimated from the slope of the transition. Data taken at 500 mK.

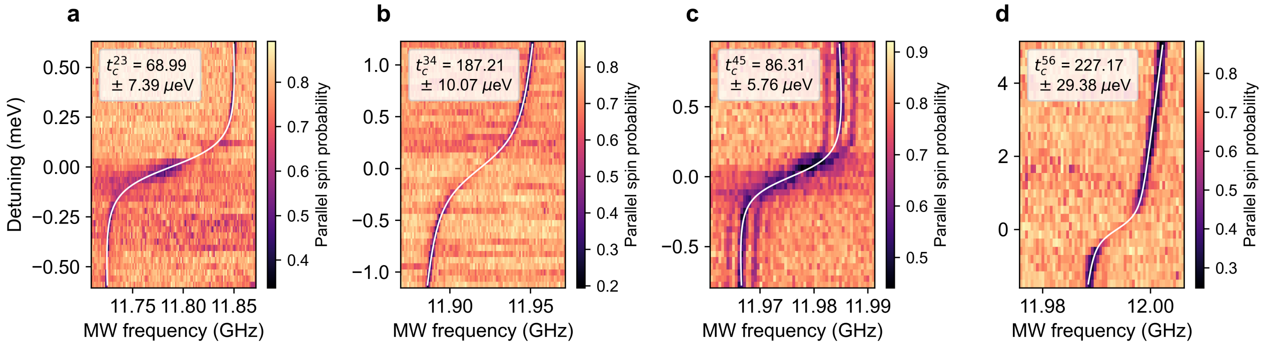

Supplementary Figure 3. **Fitting the tunnel couplings.** Measured spin probability as a function of applied microwave frequency as the interdot detuning is scanned across the interdot transition for each double quantum dot. From the transition, the interdot tunnel coupling is extracted (see text).

III. GATE VOLTAGE CONDITIONS DURING SHUTTLING

The pulse sequence schematics for bucket-brigade (BB) shuttling in Figure 2d of the main text and for conveyor-mode (CV) shuttling in Figure 3a and 3b of the main text are simplified representations of the respective shuttling methods. Supplementary Figure 4 shows the actual voltage signals applied in the experiments for BB shuttling between dot 2 and 5 (Figure 2e of the main text) and CV shuttling during IRB (Figure 4 of the main text), transporting the spin forth and back once. In bucket-brigade, we employ a 2 ns detuning ramp time during a 4 ns barrier gate pulse, as we systematically observe larger phase-flip probabilities with 1 ns ramps than with 2 ns ramps. Presumably the 1 ns ramp time in combination with the short wait time between ramps does not allow the pulse to reach full amplitude given the waveform generator bandwidth. After the electron has been shuttled through a quantum dot, the dot potential is farther detuned during the next transitions in order to prevent transferring the charge backwards. The total wait time per quantum dot is 6 ns. For the CV measurement, two-tone sinusoidal signals are applied with different amplitudes to gates in different metal layers in order to compensate for the uneven lever arms. Instead of P2, we use the virtual plunger VP2 to avoid changing the electron occupation in quantum dot 1. The spin is transported from approximately

underneath gate P5 to P2 and back. To avoid activating the exchange interaction with the reference spin in dot 1, we shuttle up to a $\frac{\pi}{5}$ conveyor phase offset from under P2. The location of the spin under gate P5 is then determined by the integer 4 ns conveyor time at a frequency of 300 MHz. The DC voltage conditions are given in Supplementary Data Table I. In the conveyor case, these values include pulsed voltage offsets during the entire duration of conveyor operation, as we need different DC voltages while shuttling than during initialization and readout.

To determine the DC voltages for conveyor operation, we usually start by tuning the predefined static quantum dots. We then increase the DC voltages of the barrier gates, while lowering the DC voltages on the plunger gates, such that the potential landscape is flattened (starting from having six local potential minima forming six dots), compensating for disorder in the spatial variation of the conduction band minimum. This disorder in the background potential can have various origins. First, local lattice strain induced by the deposition and cooldown of the gate electrodes gives rise to local modulations of the conduction band minimum. Second, charge defects in the gate dielectrics can lead to electrostatic disorder in the shuttling channel. Third, non-uniformity in the fabrication of the gate electrodes can lead to variations in the gate lever arms. In order to assess the smoothness of the potential landscape achieved by compensating disorder with the DC voltages, we track the spin resonance frequency during shuttling using EDSR spectroscopy. Once a smooth variation of the spin resonance frequency is reached (as opposed to the stair-case like variation for bucket-brigade shuttling), we further improve the shuttling fidelity by either manually or algorithmically fine-tuning the DC voltages to minimize the Ramsey phase-flip probability during back-and-forth shuttling.

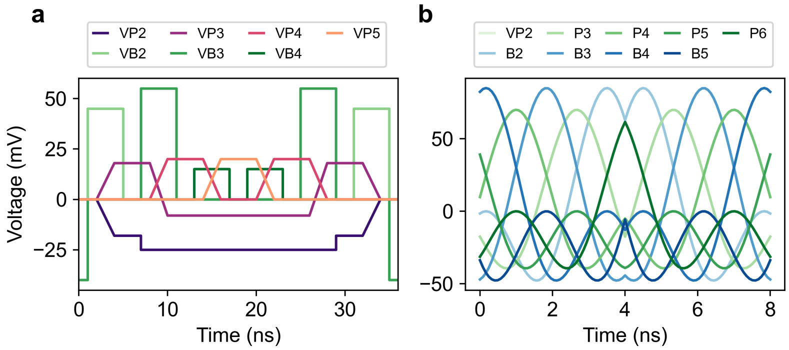

Supplementary Figure 4. **Applied shuttling voltages.** a) Pulses for BB shuttling from dot 5 to 2 and back. b) Sinusoidal signals for two-tone CV shuttling from approximately under gate P2 to P5 and back at a main conveyor frequency of 300 MHz.

Supplementary Data Table I. DC voltages applied to the shuttling channel gates during bucket-brigade (BB) and conveyor-mode (CV) shuttling.

	BB	CV
VP2	1212.82 mV	1095.91 mV
B2	771.68 mV	895.59 mV
P3	744.92 mV	626.80 mV
B3	882.29 mV	1009.31 mV
P4	883.65 mV	789.99 mV
B4	968.59 mV	1060.28 mV
P5	642.24 mV	581.91 mV
B5	1135.03 mV	1170.99 mV
P6	670.47 mV	622.13 mV
B6	763.57 mV	692.92 mV

IV. CONSIDERATIONS ON NOISE SOURCES

In the case of BB shuttling we identify several noise sources that can conceivably lead to shuttling fidelities below the limit set by dephasing in a static dot, in other words to consider phase-flip mechanims that are intrinsically connected to the tunneling events. First, in case the charge transitions are not perfectly adiabatic with respect to the tunnel couplings, the uncertainty in the moment of charge transfer leads to dephasing, as the dots have a different Zeeman splitting. However, given the measured tunnel couplings (Supplementary Figure 3), the pulse ramp times used, and the simulations in Supplementary Figure 5, it is unlikely

that diabatic transitions will dominate the shuttling performance in the absence of noise. This is further substantiated by the fact that an increasing ramp time lowers the shuttling fidelity, as shown in Figure 2f of the main text; in case diabatic transitions were caused by the pulse flanks, the reverse trend would have been observed. However, it is known that high-frequency noise can also cause diabatic transitions [5]. In this case, slower ramps will give noise more time to cause diabatic transitions and the experimentally observed trend agrees with the predicted one.

A next possible mechanism is spin-flip tunneling, which can occur due to the intrinsic spin-orbit interaction (SOI) or an effective SOI from the micromagnet when the tunnel coupling is not large enough compared to the Zeeman splitting. Spin flips caused by tunneling are also visible in shuttling experiments that test how well the spin polarization is preserved. In Si/SiGe devices with a micromagnet, [6] found a spin-flip probability 14 times smaller than the phase flip probability, suggesting other phase-flip processes are dominant during tunneling.

A plausible BB shuttling limitation, as stated in other works [7, 8], is that charge noise strongly couples to the electron when shuttling through the zero detuning point of the double quantum dots. Given the large Zeeman splitting differences between the two sites of each DQD, the resonance frequency is highly sensitive to detuning fluctuations close to zero detuning (see Supplementary Figure 3), which leads to enhanced dephasing. The BB shuttling simulations in Supplementary Section C and the experimentally observed dependence of the phase-flip probability on the ramp time corroborates this notion.

Lastly, hyperfine noise can cause spin dephasing. In this case a motional narrowing effect due to shuttling could possibly increase the coherence time, which has been experimentally observed in GaAs [9] and in ^{nat}Si/SiGe [10]. In this work, no noticeable motional narrowing was detected in bucket-brigade shuttling, though it is worth noting that the present device has only 0.08% magnetic nuclei in the quantum well versus 4.67% for natural silicon devices and 100% in GaAs.

V. SIMULATION OF DEPHASING DURING BUCKET BRIGADE SHUTTLING

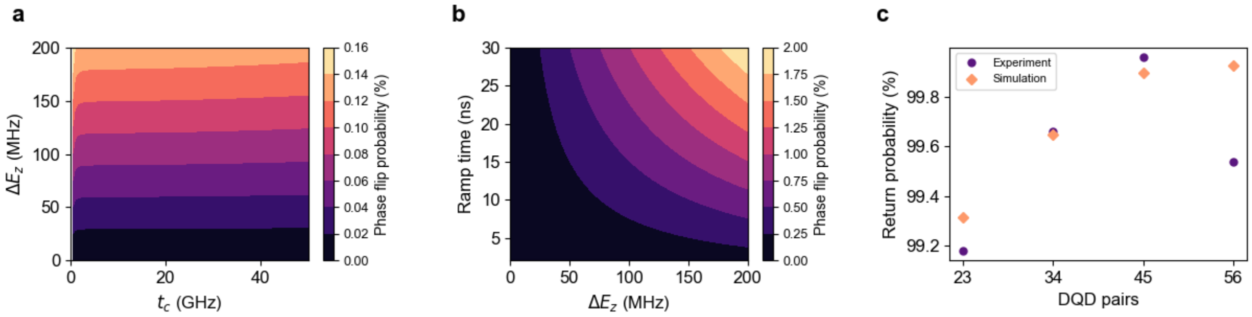

Supplementary Figure 5. **Dephasing during BB shuttling.** a) Contour plot showing the phase flip probability as a function of the tunnel coupling t_c and the Zeeman splitting difference ΔE_z between sites of a double quantum dot. b) Contour plot showing phase flip probability as a function of ΔE_z and the ramp time. c) Comparison of experimental and simulated return probabilities for different double quantum dot pairs. The parameters used for the simulation are extracted from the data in Supplementary Figure 3

In this section, we simulate the dephasing during the bucket-brigade shuttling process in the double quantum dots. Following [11], we model the charge and spin dynamics in DQDs using the Hamiltonian

$$H_s = \frac{\varepsilon}{2}\tau_z + t_c\tau_x + \frac{1}{2}g\mu_B\left[B_z\sigma_z + (b_x\sigma_x + b_z\sigma_z)\tau_z\right]. \quad (1)$$

Here, $\tau_{x,y,z}$ and $\sigma_{x,y,z}$ correspond to the Pauli matrices in the charge and spin sector, respectively. ε represents the detuning between the two dots, while t_c denotes the tunnel coupling between DQDs. $2b_x$ and $2b_z$ indicate the differences in the x and z components of the magnetic field between the DQDs, induced by the micromagnet. B_z indicates the external magnetic field (T). When the inhomogeneous magnetic fields are weak, the transverse gradient has a second-order effect on the energy splitting of the spin qubit, while the longitudinal gradient introduces a first-order effect. As a result, the Zeeman splitting E_s can be approximated as

$$E_s \simeq E_z - \frac{E_z^2 - \varepsilon^2}{2E_z\left(\Omega^2 - E_z^2\right)}(g\mu_B b_x)^2 - \frac{\varepsilon}{\Omega}g\mu_B b_z, \quad (2)$$

where $\Omega = \sqrt{\varepsilon^2 + 4t_c^2}$, and $E_z = g\mu_B B_z$.

Assuming detuning noise which fluctuates on timescales smaller than E_s, and assuming furthermore a small noise amplitude δ_ε ($\left|\frac{\partial E_s}{\partial \varepsilon}\delta_\varepsilon\right| \ll E_s$), we can estimate the dephasing rate Γ_s for the spin qubit by employing time-independent perturbation theory, as

$$\begin{aligned}\Gamma_s &= \left[\mathrm{Var}\left(\frac{\partial E_s}{\partial \varepsilon}\delta_\varepsilon + \frac{1}{2}\frac{\partial^2 E_s}{\partial \varepsilon^2}\delta_\varepsilon^2\right)/2\right]^{1/2} \\ &= \left[\gamma_s^{(1)^2} + \gamma_s^{(2)^2}\right]^{1/2},\end{aligned} \tag{3}$$

where $\gamma_s^{(1)} = \gamma_\varepsilon \frac{\partial_\varepsilon E_s}{\partial \varepsilon}, \gamma_s^{(2)} = \gamma_\varepsilon^2 \frac{\partial_\varepsilon^2 E_s}{\partial \varepsilon^2}$, and $\gamma_\varepsilon = \sigma_\varepsilon/\sqrt{2}$, where σ_ε is the standard deviation of the fluctuations δ_ε.

Assuming a completely adiabatic shuttling process, the phase flip probability due to detuning noise accumulated during shuttling can then be calculated as

$$1 - F_p = 1 - \exp\left(\int_0^{t_r} -\Gamma_s(t)t\, dt\right), \tag{4}$$

where t_r is the detuning ramp time. We note that this expression assumes that the noise fluctuates during the shuttling rounds, as can be expected from motional narrowing effects [10]. This is also consistent with the experimentally observed exponential decay of spin coherence in Ramsey-style shuttling experiments.

Supplementary Figure 5a shows the simulated phase flip probability per shuttle hop as a function of t_c and b_z, calculated with $E_z = 48.8\,\mu\text{eV}$, $g\mu_B b_x = 0\,\mu\text{eV}$, and $t_r = 2\,\text{ns}$.

Supplementary Figure 5b shows the same simulation but as a function of tunnel coupling t_c and ramp time t_r, calculated with $E_z = 48.4\,\mu\text{eV}$, $g\mu_B b_x = 0\,\mu\text{eV}$, and $t_c = 124.2\,\mu\text{eV}$. Similar to the experimental data shown in Figure 2f of the main text, the phase flip probability increases as the ramp time increases.

Supplementary Figure 5c shows a comparison of the return probability obtained from the experiment and the simulation. For the latter, the tunnel couplings and Zeeman splitting differences are extracted from Supplementary Figure 3. Furthermore, $\gamma_\varepsilon = 6.5\,\mu\text{eV}$ is used, based on the average T_2^* and $1/\Gamma_s$, taking into account the difference in measurement time. Except for dot 5-6, the phase flip probability tends to decrease as b_z decreases in both simulation and experiment.

VI. BUCKET-BRIGADE SHUTTLING FROM QUANTUM DOT 2 TO 6

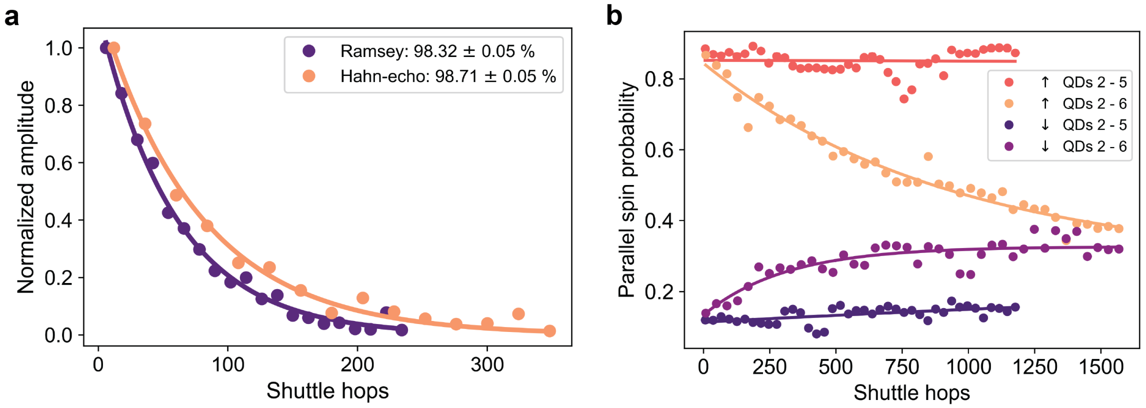

Supplementary Figure 6. BB shuttling between quantum dot 2 and 6. a) Normalized amplitudes of Ramsey- and Hahn-echo-style measurements during bucket-brigade shuttling between QD2 and QD6. b) Spin relaxation during bucket-brigade shuttling between dot 2 and 5 and between dot 2 and 6. Including quantum dot 6 in the shuttling trajectory leads to a significant increase in the relaxation rate. The solid lines are exponential fits to the data.

In Supplementary Figure 6a the decay of Ramsey and Hahn-echo fringes during BB shuttling between dot 2 and dot 6 is presented. Bucket brigade shuttling across the entire array is therefore possible. Nonetheless, including dot 6 in the BB chain considerably decreases the shuttling performance. In Supplementary Figure 6b the spin polarization as a function of the number of shuttle hops is shown. The relaxation rate is very small when the spin is shuttled between quantum dot 2 and 5. When shuttling between quantum dot 2 and 6, the relaxation rate increases dramatically. We have furthermore observed a low lever arm of gate B5 and a small charging energy in dot 5. We speculate that the small orbital energy could induce diabatic charge excitations.

Given the artificial spin-orbit interaction from the micromagnet, this would affect not only the spin dephasing rate (phase flips) but also the spin relaxation rate (spin flips) during shuttling. We point out that a comparison of Supplementary Figure 6b to the dot 5-6 results in Fig. 2b and 2c of the main text is difficult. Not only does the former include barrier pulses during shuttling, the VB5 voltage operation point during the 5-6 detuning pulse is 20 mV higher compared to the double dot shuttling in Fig. 2 of the main text.

VII. IDLING SITE FOR DOUBLE DOT BUCKET-BRIGADE SHUTTLING

The idling operation in Fig. 2a of the main text is performed in different dots for the Ramsey and Hahn-echo sequences. Ideally, the idle time would be equally divided between the two sites in order to also eliminate the effect of any T_2^* difference in the dots. To evaluate its impact, we assume dephasing with Gaussian decay in both sites of the double quantum dot and simulate the difference in the phase-flip probability when performing the final idling time in different dots. This difference decreases linearly with the number of shuttle hops, as the idling time necessarily becomes shorter. From our simulation, the difference is limited to -0.04%, -0.02% and 0.02% per hop for pair 2-3, 3-4 and 4-5, respectively, which falls within the standard deviation to the fit in Fig. 2b of the main text. The negative (positive) sign of the difference, refers to a slight over(under)estimation of the phase-flip probability. We can therefore conclude that, despite the non-ideal implementation of the idling time in Fig. 2a of the main text, there is no significant effect on the extracted phase-flip probabilities.

VIII. VALLEY SPLITTING

Magnetospectroscopy measurements of the two-electron singlet-triplet energy splittings E_{ST} in this device were performed and reported by [2], albeit in a different cooldown and gate voltage configuration than these shuttling experiments. The estimated E_{ST} values for all quantum dots are indicated in Supplementary Data Table II. E_{ST} is a lower bound for the single-particle valley splitting E_v in strongly confined quantum dots [12] and is also the relevant metric for the size of the Pauli-spin-blockade readout window. For our shuttling experiments, in each quantum dot the singlet-triplet energy splitting is significantly larger than the Zeeman splitting. Dephasing at spin valley hot-spots is therefore suppressed.

Supplementary Data Table II. E_{ST}, as reported by [2], for each quantum dot in this device, which serves as a lower bound for the single-particle valley splitting.

QD 1	QD 2	QD 3	QD 4	QD 5	QD 6
208 µeV	174 µeV	276 µeV	208 µeV	243 µeV	278 µeV

IX. DEPHASING TIME DURING CONVEYOR SHUTTLING

The data represented in Figures 3d and 3e of the main text show the phase flip probabilities for conventional and two-tone conveyor-mode shuttling per interdot distance d. As the transfer speed is varied, the total duration between initialization and readout is not identical for each data point. To investigate the effect of the conveyor shuttling process itself, we can fit the dephasing time of the spin during shuttling. Supplementary Figure 7 shows this dephasing time when conveyor-mode shuttling during the wait times of Ramsey and Hahn-echo sequences. For conveyor frequencies between 10 and 100 MHz, the average dephasing time during shuttling is 2.49 µs for the conventional, and 2.69 µs for the two-tone conveyor. In the case of a static conveyor potential shown in Figure 1d of the main text, the dephasing time is 1.45 µs on average. This increase in dephasing time can likely be explained by motional narrowing. We therefore conclude that, different from the bucket-brigade results, the motion of the conveyor-mode potential does not limit the transfer fidelity, and probably enhances spin coherence.

X. DATA GAP FOR THE 100-160 MHZ CONVEYOR FREQUENCY BAND.

Here we comment on the missing data points in Fig. 3d of the main text for conveyor frequencies between 100 MHz and 160 MHz. In this frequency range, we do not observe Ramsey or Hahn-echo oscillations for any number of conveyor shuttle rounds. An arguably similar loss of charge shuttle fidelity at specific conveyor frequencies was observed in [13], where the authors speculate the origin to be a resonance with a charge defect. We note that in our device the RF reflectometry circuits have resonances located at 135 MHz and 142 MHz. A conveyor operating at these frequencies could therefore lead to a loss of

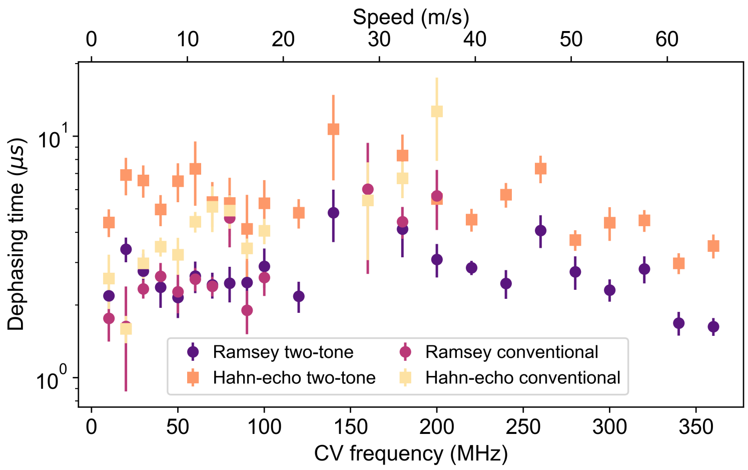

Supplementary Figure 7. **Coherence during conveyor shuttling.** Dephasing time and Hahn-echo decay time in a moving conveyor, both for the conventional and two-tone conveyor.

readout. However, we observe that the frequency band where shuttling fails is much broader than the RF resonances and that two-tone conveyor shuttling is not affected.

XI. TWO-TONE CONVEYOR SHUTTLING FIDELITY WITH DRIVEN EXTERNAL ELECTROMAGNET

After completion of the shuttling measurements, we found that, for a spin in a static conveyor, up to twice longer T_2^* values are obtained with the superconducting magnet in persistent mode, reducing external field fluctuations. This suggests that global magnetic-field fluctuations were likely affecting the spin-shuttling fidelities. This is confirmed by Supplementary Figure 8a and b, where a significant increase in the coherence time during conveyor-mode shuttling is observed when the state of the magnet is persistent. For comparison with Fig. 4b in the main text, we show shuttling IRB with the magnet in driven mode in Supplementary Figure 8c. The shuttling frequency and time per round are identical, though the shuttling gate comprises 23 instead of 24 shuttle rounds.

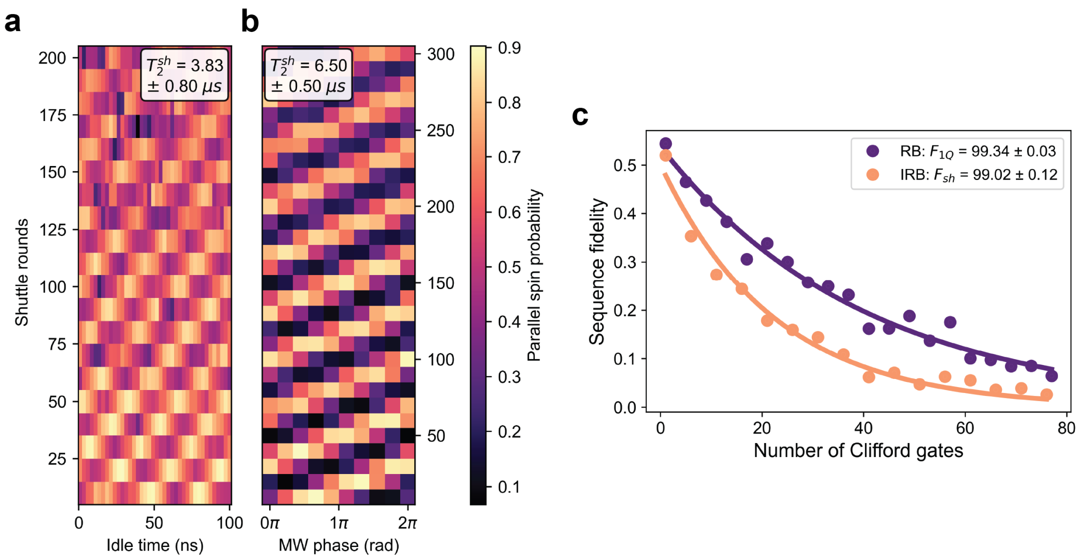

Supplementary Figure 8. **Coherence time depends on the operating mode of the superconducting magnet.** a,b) A single electron is shuttled back-and-forth using a two-tone conveyor at a main frequency of 300 MHz and with a 4 ns one-way shuttling time. a) Ramsey-like measurement, as used for Fig. 3d of the main text, where the idling time refers to a variable wait time after shuttling a number of rounds and before readout. The superconducting magnet is in driven mode. b) Similar Ramsey-like measurement, where the idling time is replaced with a virtual Z gate with variable phase before the final X_{90}. In this case, the superconducting magnet is in persistent mode. The insets indicate an estimated coherence time during shuttling, extracted from fitting the decay in oscillation amplitude with shuttle rounds using an exponential function. c) Conveyor shuttling with the superconducting magnet in driven mode. Interleaved randomized benchmarking with 23 shuttle rounds yields a shuttle fidelity of 99% for an effective 10 μm shuttling distance.

XII. TWO-TONE CONVEYOR SHUTTLING FIDELITY WITH EQUAL DC VOLTAGES

In Figure 3c of the main text we demonstrate that conveyor-mode shuttling with equal DC offsets is possible, though requiring higher pulse amplitudes. Here we show interleaved randomized benchmarking of shuttling using a two-tone conveyor at 80 MHz while emulating a lack of individual gate control. To do so, the conveyor amplitude is set to 90 mV for each gate, regardless of the gate layer. This is with the exception of VP2 (70 mV), as needed to keep the reference electron confined in dot 1. We apply pulse offsets such that the barrier gates B2, B3, B4 and B5 in the conveyor are set to a DC voltage of 900 mV, and the plunger gates P3, P4, P5 and P6 are set to a DC voltage of 710 mV while shuttling. Gates on the edges of the device were exempted, as they are crucial not only for the initialization and readout but also for the transition of the single electron into the conveyor. Moreover, it is imperative to avoid losing the shuttled electron to the reservoirs. In Supplementary Figure 9 the fidelity for shuttling over a distance of 432 nm without individual gate control is determined to be 99.18 ± 0.19 %. Extrapolating to 10 μm from the case with individual DC control, this amounts to a fidelity of 65.08 ± 2.90 %.

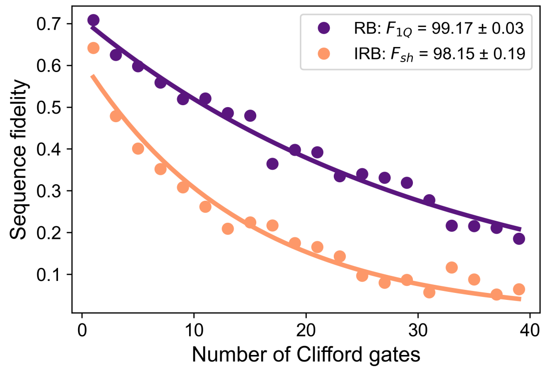

Supplementary Figure 9. **Conveyor shuttling IRB with equal DC voltages.** Interleaved randomized benchmarking with equal DC voltages applied to all the channel gates in the same layer. Here, the shuttling gate comprises a single shuttle round and a phase gate.

XIII. REQUIRED CONVEYOR AMPLITUDE CORRECTED FOR AWG FILTER

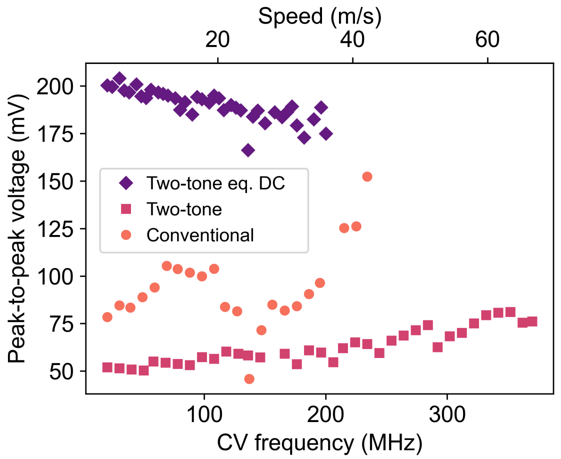

Supplementary Figure 10. **Required peak-to-peak pulse voltage after AWG filter.** Minimal peak-to-peak gate voltage required for successful charge transfer by conveyor shuttling, accounting for the filter function built-in to the AWG.

Whereas Figure 3c of the main text takes into account the attenuation in the transmission lines, it does not consider the output filter of the waveform generator. In Supplementary Figure 10 we correct for the phase and amplitude distortion of the applied sine signals using the measured AWG filter function. Note that in the two-tone case, the filter response is different for both sine waves, leading to an alteration of the shape of the traveling wave potential with increasing conveyor frequency. Therefore, here we indicate the peak-to-peak voltage of the applied signals. It is not clear to us why the required peak-to-peak voltage for successful charge transfer (slightly) decreases with conveyor frequency for the two-tone implementation with equal DC voltages. If anything, we were a priori expecting that higher amplitudes might be needed for higher conveyor frequencies, as seen in the other two cases shown. As stated in the main text, smaller amplitudes are required for the two-tone conveyor than for the conventional conveyor. This is what we were expecting given that the potential barriers surrounding a moving dot are wider for the two-tone conveyor (see also the diagrams in Fig. 3a and 3b of the main text), and an electron is therefore less likely to escape from the potential minimum in which it is meant to travel.

XIV. MICROMAGNET SIMULATION

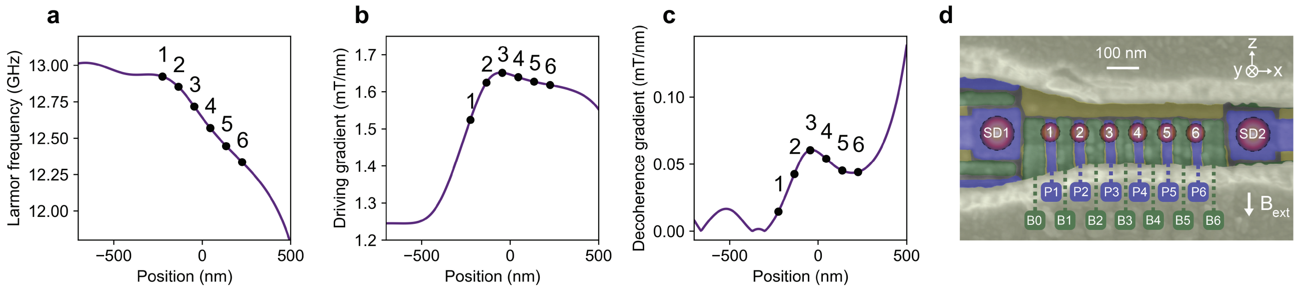

Supplementary Figure 11. **Micromagnet simulation.** a) Simulated qubit frequencies along the array at an external magnetic field of 0.26 T, with the target qubit positions along the x direction indicated. The micromagnet is magnetized along the z direction with a magnetization vector of M = (0, 0, -1.5) T. The experimentally observed Larmor frequency trend in Figure 1c of the main text, though also monotonic, is opposite to the simulated one. Simulations with reasonably altered parameter values of the magnet geometry can produce a parabolic-like trend, similar to experimental observations in [14], though a Larmor frequency trend akin to the experimental one is not found. Nonetheless, the experimentally observed frequency trend has been measured consistently in multiple samples. b) Simulated transverse driving gradient of the micromagnet c) Simulated decoherence gradient of the micromagnet. All simulations were performed using the python package magpylib. d) False-colored SEM image of a nominally identical device, indicating the frame of reference and the applied magnetic field direction.

XV. MEASUREMENT SET-UP

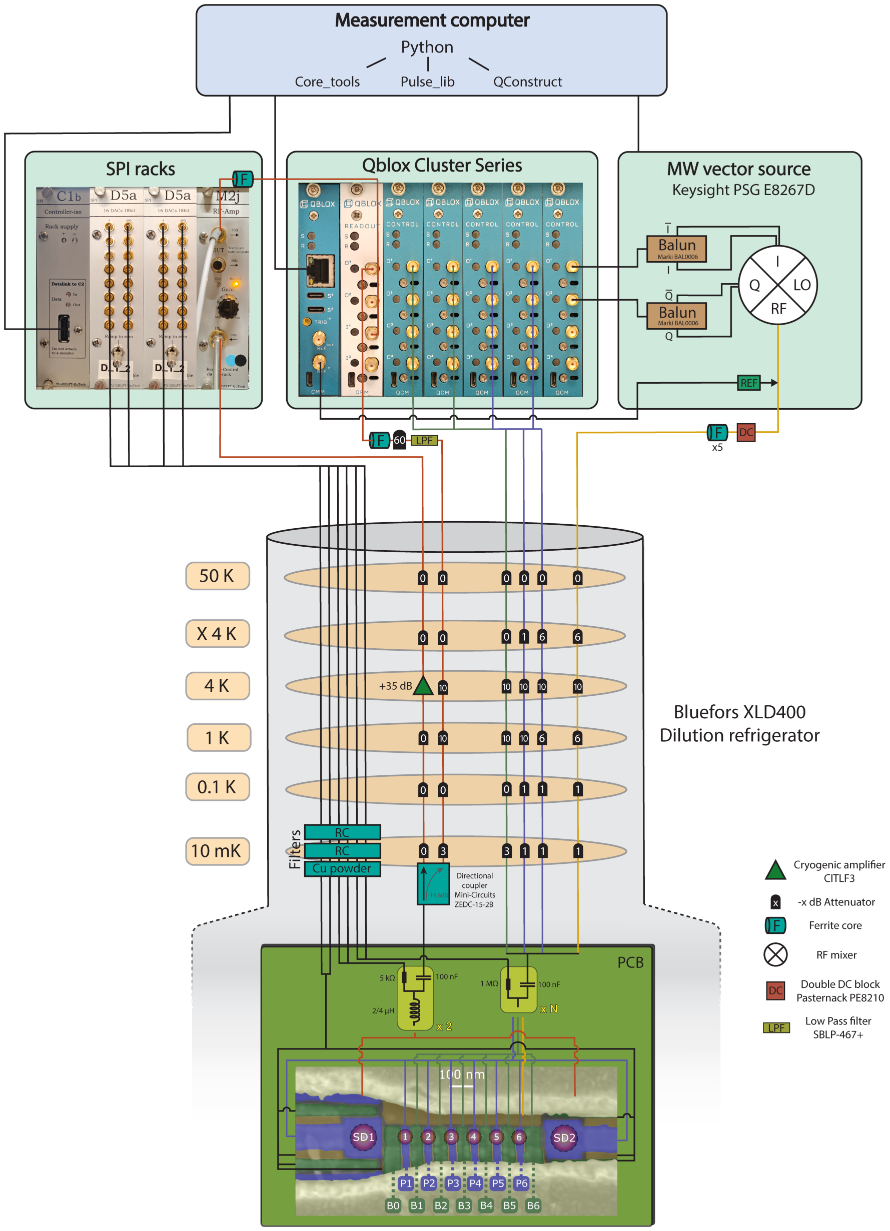

Supplementary Figure 12. **Schematic of the experimental setup.** The experiments were executed using three main pieces of electronics (shown in green). The SPI rack, powered by batteries and equipped with gyrators, houses two sets of in-house built DACs. They provide low-noise DC voltages that pass through RC and copper powder filters, before arriving at the PCB. Baseband pulses below 400 MHz are applied using QCM modules in the Qblox Cluster Series. The pulses are attenuated at different stages in the Bluefors XLD400 dilution refrigerator, amounting to -23 or -24 dB attenuation in total. Bias tees on the PCB with an RC time constant of 100 ms combine the pulses with the DC voltages. All plunger and barrier gates in the channel, as well as the sensing dot plungers, are equipped with these bias tees. For readout, RF reflectometry is implemented on both sensing dots. RF signals generated by the Qblox QRM module are sent through a ferrite core, a -60 dB attenuator and a low pass filter at room temperature. After -23 dB additional attenuation in the dilution refrigerator, the RF signal is split in two and directed to a bias tee in series with an off-chip superconducting NbTiN inductor for each sensing dot. The inductor is wire-bonded directly to a reservoir accumulation gate neighboring the sensing dot. The reflected RF signal passes through the directional coupler and is amplified at the 4 K stage using a Cosmic Microwave Technology CITLF3 cryogenic amplifier. It is then amplified again at room temperature using an in-house built M2j low-noise amplifier, after which IQ demodulation is performed by the QRM. We use a Keysight PSG E8267D microwave vector source for EDSR driving. The I and Q input signals are generated by a Qblox QCM and differentiated using Marki BAL006 Baluns. Ferrite cores and a double DC block are added to the microwave line. After a total of -24 dB attenuation in the refrigerator, the MW signal is routed via a coplanar waveguide on the chip to the top screening gate.

XVI. DATA ANALYSIS OF RAMSEY- AND HAHN-ECHO-LIKE SHUTTLING MEASUREMENTS

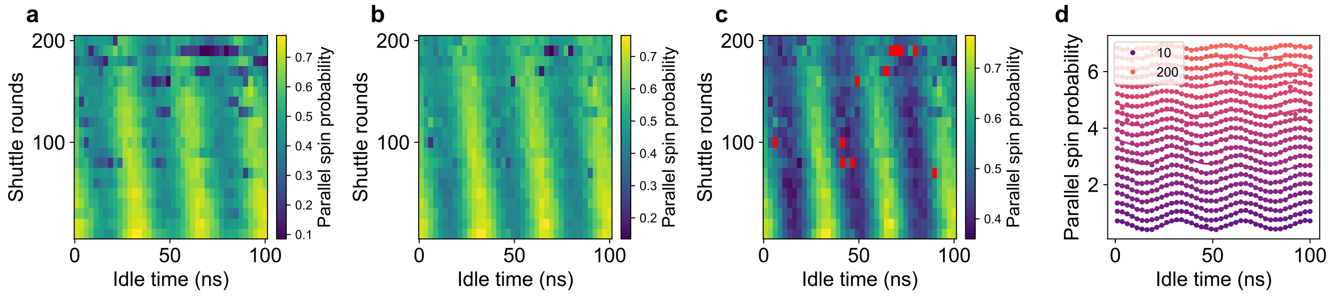

Supplementary Figure 13. **Data analysis for Fig. 3d of the main text.** a) Raw data example of a Hahn-echo-like measurement used in Fig. 3d of the main text while shuttling a number of rounds (back-and-forth from approximately under gate P2 to under gate P5) with a two-tone conveyor having a main frequency of 280 MHz. b) The Hahn-echo-like measurement from a) after dynamical rethresholding by double gaussian fitting for each point in the 2D plot. c) The Hahn-echo-like measurement from b) after setting a global minimum probability to remove the outliers (red) that were not recovered with dynamical rethresholding. d) Fitting the parallel spin probability of the data in c) with a cosine function for each number of shuttle rounds in order to extract the decay in amplitude. Each line is offset by 1/3 for clarity.

---

[1] Lawrie, W. I. L. *et al.* Quantum dot arrays in silicon and germanium. *Applied Physics Letters* **116**, 080501 (2020).

[2] Degli Esposti, D. *et al.* Low disorder and high valley splitting in silicon. *npj Quantum Information* **10**, 1–9 (2024).

[3] Degli Esposti, D. *et al.* Wafer-scale low-disorder 2DEG in ^{28}Si/SiGe without an epitaxial Si cap. *Applied Physics Letters* **120**, 184003 (2022).

[4] Feng, M. *et al.* Control of dephasing in spin qubits during coherent transport in silicon. *Physical Review B* **107**, 085427 (2023).

[5] Krzywda, J. A. & Cywiński, Ł. Adiabatic electron charge transfer between two quantum dots in presence of $1/f$ noise. *Physical Review B* **101**, 035303 (2020).

[6] Noiri, A. *et al.* A shuttling-based two-qubit logic gate for linking distant silicon quantum processors. *Nature Communications* **13**, 5740 (2022).

[7] Yoneda, J. *et al.* Coherent spin qubit transport in silicon. *Nature Communications* **12**, 4114 (2021).

[8] van Riggelen-Doelman, F. *et al.* Coherent spin qubit shuttling through germanium quantum dots. *Nature Communications* **15**, 5716 (2024).

[9] Mortemousque, P.-A. *et al.* Enhanced spin coherence while displacing electron in a two-dimensional array of quantum dots. *PRX Quantum* **2**, 030331 (2021).

[10] Struck, T. *et al.* Spin-EPR-pair separation by conveyor-mode single electron shuttling in Si/SiGe. *Nature Communications* **15**, 1325 (2024).

[11] Benito, M. *et al.* Electric-field control and noise protection of the flopping-mode spin qubit. *Physical Review B* **100**, 125430 (2019).

[12] Ercan, H. E., Coppersmith, S. N. & Friesen, M. Strong electron-electron interactions in Si/SiGe quantum dots. *Physical Review B* **104**, 235302 (2021).

[13] Seidler, I. *et al.* Conveyor-mode single-electron shuttling in Si/SiGe for a scalable quantum computing architecture. *npj Quantum Information* **8**, 1–7 (2022).

[14] Philips, S. G. J. *et al.* Universal control of a six-qubit quantum processor in silicon. *Nature* **609**, 919–924 (2022).
